# Metamaterial array based meander line planar antenna for cube satellite communication

**DOI:** 10.1038/s41598-021-93537-6

**Published:** 2021-07-08

**Authors:** Touhidul Alam, Ali F. Almutairi, Md Samsuzzaman, Mengu Cho, Mohammad Tariqul Islam

**Affiliations:** 1 Space Science Centre (ANGKASA), Institute of Climate Change (IPI), Universiti Kebangsaan Malaysia, 43600 Bangi, Selangor Malaysia; 2 Electrical Engineering Department, Kuwait University, 13060 Kuwait, Kuwait; 3 Department of Computer and Communication Engineering, Faculty of Computer Science and Engineering, Patuakhali Science and Technology University, Dhaka, Bangladesh; 4 Laboratory of Spacecraft Environment Interaction Engineering (LaSEINE), Kyushu Institute of Technology, Fukuoka, 804-8550 Japan; 5 Department of Electrical, Electronic and Systems Engineering, Faculty of Engineering and Built Environment, Universiti Kebangsaan Malaysia, 43600 Bangi, Selangor Malaysia

**Keywords:** Electrical and electronic engineering, Metamaterials

## Abstract

This research article presents a design and performance analysis of a metamaterial inspired ultra-high frequency (UHF) compact planar patch antenna for the CubeSat communication system that could be smoothly integrated with commercially available 2U Cube Satellite structure and onboard subsystem. The proposed antenna consists of two layers, one is two different width meander line antenna patch with partial ground plane and another layer is 3 × 2 near-zero-indexed metamaterial (NZIM) metamaterial array structure with ground plane. The NZIM array layer has been utilized to minimize the coupling effect with Cube Satellite structure and improve the frequency stability with enhanced antenna gain and efficiency. The fabricated antenna can operate within the lower UHF frequency band of 443.5–455 MHz. with an average peak gain of 2.5 dB. The designed antenna impedance stability characteristic has been explored after integration with the 2U Cube Satellite body layout. Besides, the antenna communication performance has been verified using 2U Cube Satellite free space path loss investigation. Small antenna volume with trade-off between the antenna size and performance are the key advantages of the proposed design, as the antenna occupies only 80 × 40 × 3.35 mm^3^ space of the 2U Cube Satellite body structure and the geometrical parameters can be designed to provide the best performance between 449 and 468.5 MHz.

## Introduction

Cube satellite missions have increased dramatically in both research and commercial sectors due to economic affordability and short building period. Cube satellites are tiny cube-shaped small satellites with Unit (U) dimensions of 10 × 10 × 10 cm^3^ and a mass less than 1.33 kg per U. The cubic structure encompasses an enclosed aluminum box with solar cells fitted to the outside walls. The antenna is a crucial element of the satellite communication system. The development of a low profile with efficient antenna performance is highly desirable for the Cube Satellite communication system. However, inherently inverse proportionality between antenna performance and size is the big challenge to design Cube satellite antenna. Maintaining good performance with specific Cube satellite requirements represent a major mechanical and RF challenge. Mutual coupling between the antenna and other components that can degrade antenna performances is one of the key challenges of the small satellite antenna. Over the last decade, several types of antennas have been developed for the small satellite communication system, which can be summarized in two categories: deployable antenna and non-deployable antenna. Both types of antennas have been developed by considering the size and weight of the Cube satellite, which are the most vital factors that have a profound influence on antenna type and design^1^.

Wire antennas are widely used a deployable antenna in small satellites at high frequency (HF), VHF, and UHF applications. Monopole, dipoles, Yagi-Uda arrays and helical antennas are different types of wire antennas, which are typically placed on the outer face of the small satellite structure to facilitate space for other electronic components. The adverse fact of this type of antenna is that they require different deployment mechanism. But the mechanical deployment of this type of antenna is stimulating, and which might be increased the chance of mission failure^2, 3^. In contrast to the deployable antenna, non-deployable antennas like, patch antennas provide a modest solution to this issue with better mission reliability. However, this type of antenna has a frequency shifting issue due to a non-infinite ground plane and the effect of a metallic satellite body. Planar antennas have gain special attention for the small satellite communication system, owing to their low profile and ease of fabrication^4–6^. However, the size of the lower UHF antenna becomes larger in size, which grabs a large volume of the CubeSat body surface and makes them difficult to place adequate solar cells. To address these issues, the high dielectric substrate (εr = 10.2) based patch antenna has been developed for the USUSAT nanosatellite mission^7^. The antenna was developed for 450 MHz uplink data of Ionospheric Observation Nanosat Formation (ION-F) constellation. After that, lots of patch antennas have been studied for the nanosatellite communication system^8, 9^. In^10^, a UHF 433 MHz printed patch antenna has been developed, which was printed on 51 mm × 28 mm FR-4 substrate material. But the major drawback of this design was low gain, only − 13 dB. Meander line technique expedites to achieve the lower band with miniature antenna dimension^11^. Nevertheless, the performance of these types of antenna degrades when fixed with the complex structure and circuitry. To enhance antenna gain and efficiency, the antenna size needs to enhance. Several antennas have been designed using the meander line structure for the UHF communication system of various nanosatellite applications, LAPAN-TUBSAT microsatellite is one of them. A meander line structured antenna has been developed for telemetry and telecommand applications of LAPAN-TUBSAT, was operated at 428–468 MHz. The antenna achieved 2.9 dBi gain with dimensions of 160 × 140  mm^2^^12^. In recent years, metamaterial inspired antenna concept has been utilized to miniaturize antenna size with maintaining effective and optimal performances^13^. So far, these metamaterial antenna designs have been performed using negative permeability, negative permittivity or double negative (DNG) metamaterial, which is used to miniaturize the antenna size due to their quasi-static resonant response^14^. Besides, another type of metamaterial has been developed to support near-infinite phase velocity and “static-like” field distributions as well as equally fascinating wave propagation properties^15^. Recently, near-zero index metamaterial loaded 400 MHz UHF antenna for nanosatellite communication system was presented^6^. The proposed antenna was consisted of a same width meander line patch and metamaterial inspired partial ground, where metamaterial structures were placed on the same plane of the ground plane. But this antenna suffers lower efficiency and lower gain. Moreover, antenna operating frequency tuning is quite difficult and do not have direct operating frequency tuning relation with a single antenna parameter. Therefore, our goal is to design an UHF antenna that can enhance antenna efficiency and gain with compact antenna dimension to reduce the risk of CubeSat mission failure in space environment.

In this paper, a lower UHF meander line antenna is designed using a near-zero metamaterial. To verify the antenna performance, the antenna has been fabricated, and the antenna's performance has been investigated. The fabricated antenna can operate within the lower UHF frequency band of 443.5–455 MHz. with an average peak gain of 2.5 dB. The designed antenna impedance stability characteristic has been explored after integration with the 2U Cube Satellite body layout. Moreover, the antenna communication performance has been verified using 2U (20 × 10 × 10 cm^3^) Cube Satellite free space path loss investigation.

## Antenna geometric layout configuration

**Figure 1:**
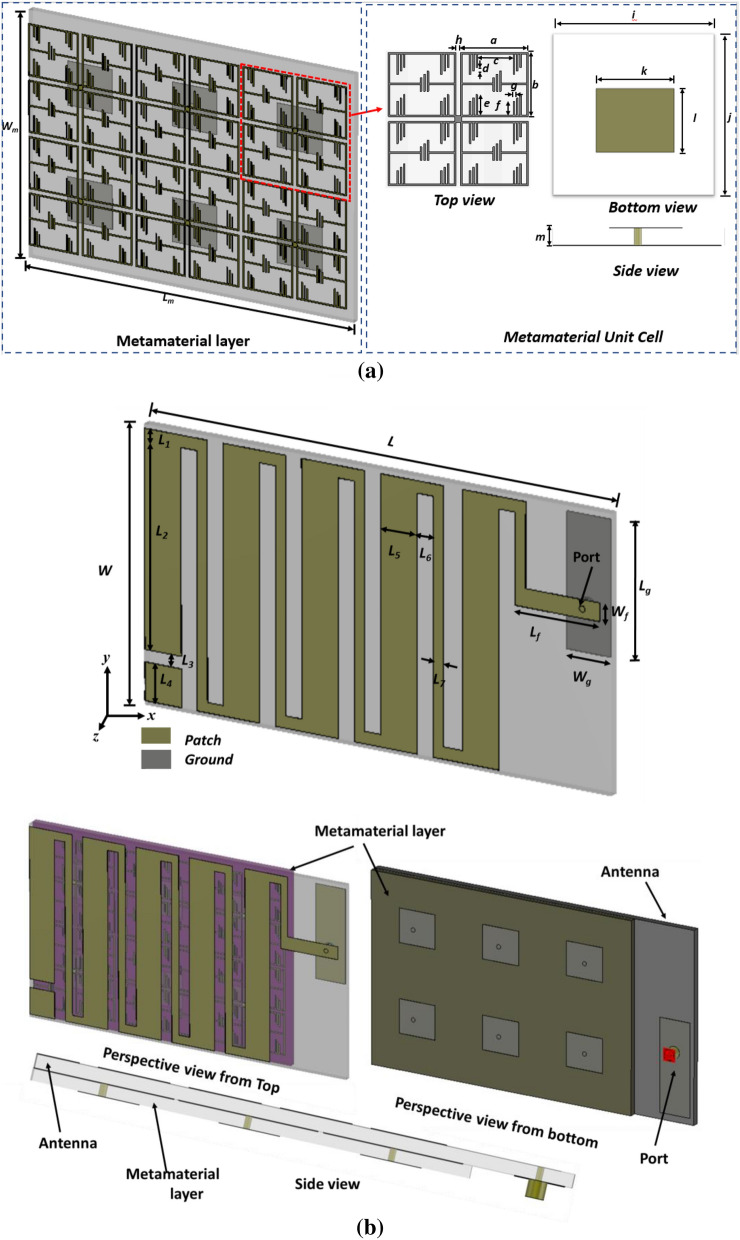
(**a**) NZIM Metamaterial layout configuration and (**b**) design layout of the optimized antenna. (CST STUDIO SUITE 2019, https://www.3ds.com/products-services/simulia/products/cst-studio-suite)^16^.

The antenna design structure of the optimized antenna and NZIM metamaterial structures are shown in Fig. 1. The proposed antenna simulation is performed by 3D electromagnetic simulator CST (Computer System Technology) microwave studio suite, 2019^16^. The antenna consists of a modified meander line patch in the top and partial ground plane on the other side of the substrate. A metamaterial layer with a 3 × 2 near-zero index metamaterial (NZIM) array is placed under the antenna, where the metamaterial structure consists of thin metallic arms and split ring resonant (SRRs) with the rectangular ground plane, which is shown in Fig. 1b. The patch antenna and metamaterial layer are designed on low loss space compatible Rogers Duroid 5880 substrate material with a height of 1.575 mm. The size of the metamaterial layer is 65 × 40 × 1.575 mm^3^. The overall optimized dimension of the antenna is 80 × 40 × 3.35 mm^3^. The initial partial grounded meander line antenna structure was designed considering conventional monopole and printed dipole mechanism and then, modified the structure to partial grounded meander line. The antenna size was chosen considering on CubeSat size constrain, conventional monopole size for 450 MHz, which is 166.5 mm × 66 mm × 1.57 mm and the size of 0.5λ printed dipole format (333.11 mm). The antenna structured was modified using fold the conductors back and forth mechanism to minimize the length of 80 × 40 × 1.57 mm^3^. Though the antenna size becomes smaller, the antenna bandwidth and radiation efficiency also decrease. As the width of the meander, line contributes to the capacitance associated with the ground plane, so the resonance frequency of the meander-lined antenna can be controlled by changing the width of the meander lines. Moreover, effective self-inductance can be modified by increasing the meander line section and thus, the lower frequency can be adjusted. The antenna size in the proposed design can be minimized by appropriate optimization of a number of folds, inter folds distance and line width. A 50-Ω coaxial direct feed is used for connecting the patch and ground plane for excitation which location is identified in the design layout in Fig. 1b. Checking the 50-Ω impedance matching initially, we have chosen the location on the right side of the meander line-starting patch and check the input impedance and surface current through the CST simulator. By tuning the position of the feed location and feed line length of 14.38 mm, we have achieved the 50-Ω impedance for the proposed antenna. The antenna is fed by a 50Ω Micro-miniature coaxial (MMCX) connector. A slot *L*_3_ has been etched from the upper arm of the meander line to tune the resonating frequency.

**Figure 2:**
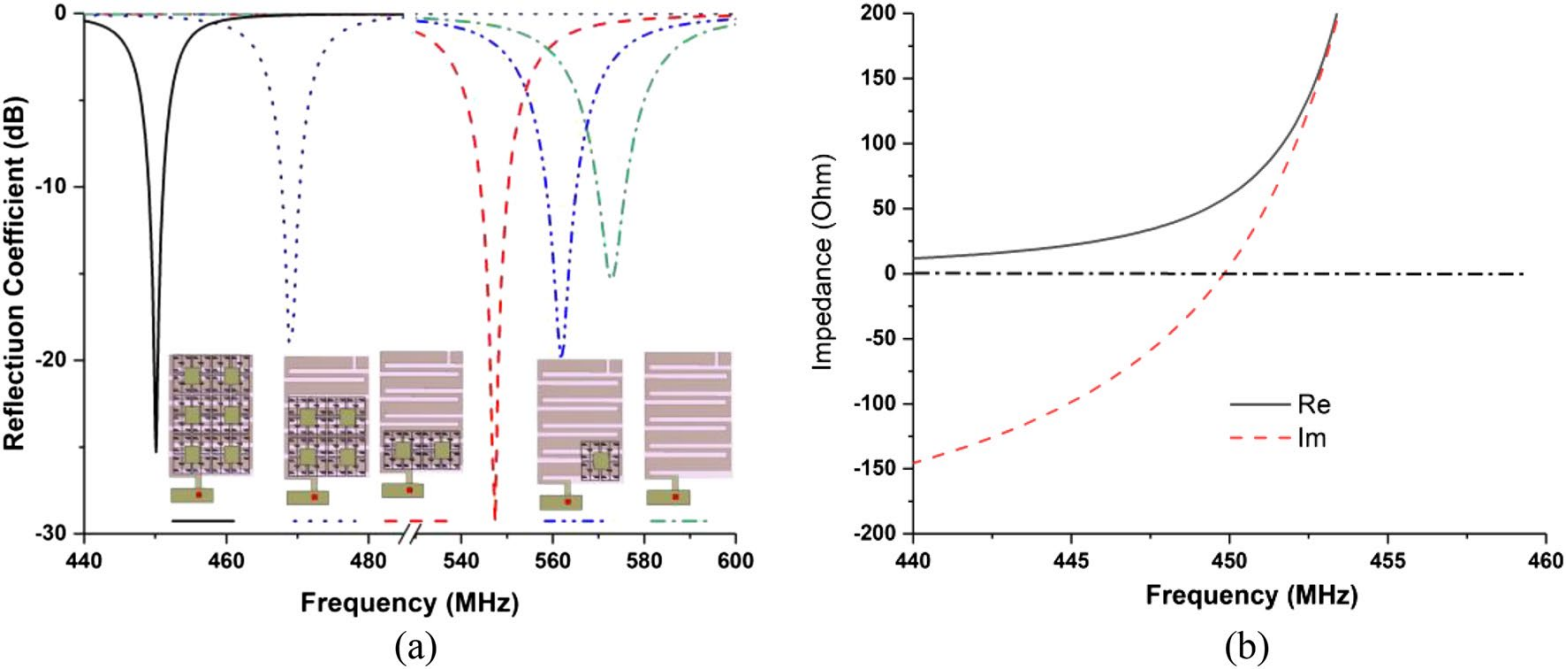
(**a**) Antenna reflection coefficient (S_11_) at various stages of the proposed design, (**b**) input impedance curve of the proposed antenna.

**Figure 3:**
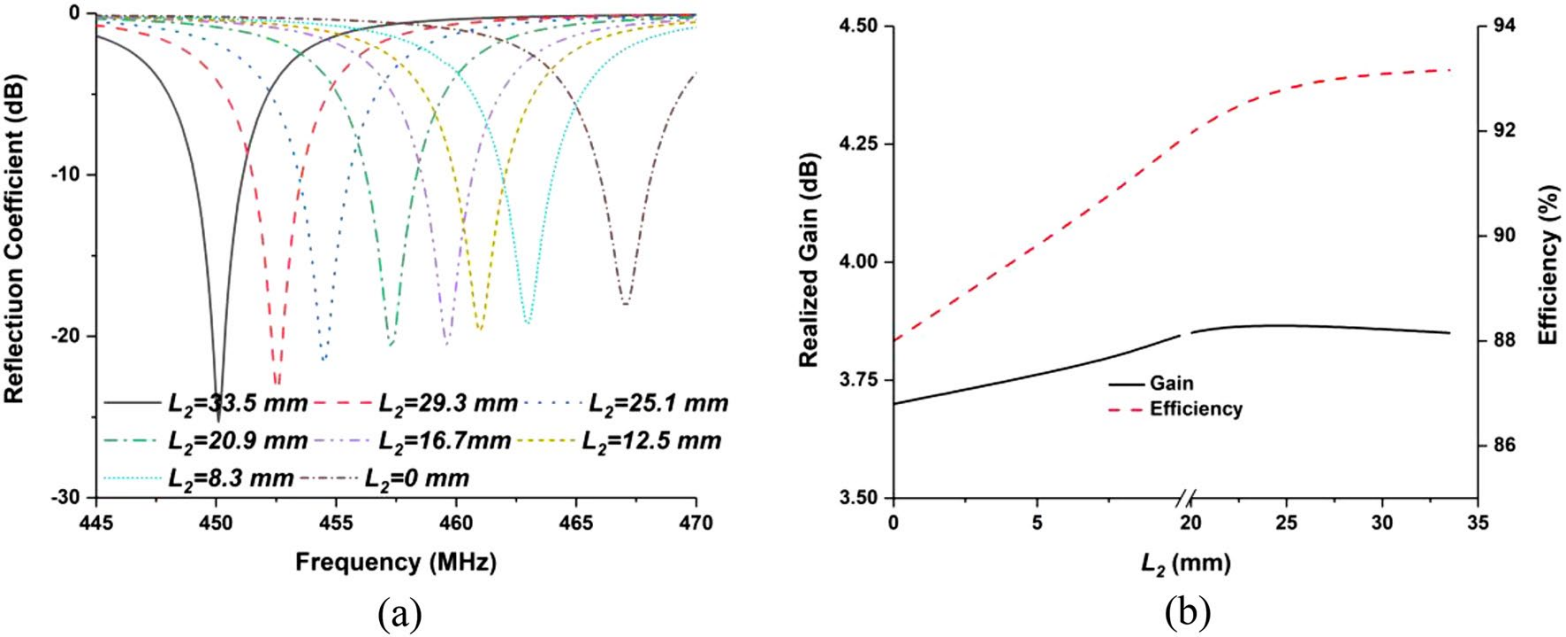
(**a**) Antenna reflection coefficient for various length of L2 and (**b**) realized gain and efficiency for different values of L2.

The design evolution of the proposed antenna is illustrated with reflection coefficient, shown in Fig. 2, where shows that the metamaterial array embedded antenna resonates at 450 MHz. To enhance antenna radiation performance, single metamaterial unit cell was placed behind ground layer of the antenna, which shifted resonating frequency downwards. To tune the antenna from 580 MHz frequency to 450 MHz, L_3_ slit was etched from the meander line structure. After that different array configuration was investigated to achieve resonant frequency at 450 MHz with optimum radiation performances, shown in Fig. 2a. Figure 2b also illustrates the input impedance curve of the proposed antenna. From this figure it can stated that, the real and imaginary (reactance) input impedance value of the proposed antenna at 450 GHz are about 50 Ω and zero, respectively. Moreover, the resonating frequency can be controlled by shifting the *L*_2_ slot position, depicted in Fig. 3. It is shown from Fig. 3a that when the slot moves upward, the *L*_2_ value decreases and the *L*_4_ value increases. The resonant frequency moves to the upper frequency by decreasing the *L*_2_ value. The optimized antenna can be tuned by changing the length of the dimension *L*_2_. The normalized Equation for the frequency tuning is proposed based on the critical parameter of *L*_2_, depicted in Eq. (1)

1 $$f\left({L}_{2}\right)=467-0.46{L}_{2}$$

The antenna can be tuned from 449 to 468 MHz by adjusting the length of the *L*_2_ value. The realized gain and total efficiency remain near constant. The final design dimension parameters of the proposed antenna are depicted in Table 1.

**Table 1:** Optimized design dimension of the finalized antenna.

Design parameters	Value (mm)	Design parameters	Value (mm)
*L*	80	*W*_*f*_	3
*L*_*1*_	2	*a*	10
*L*_2_	33.5	*b*	9.5
*L*_3_	2	*c*	5.25
*L*_4_	6.18	*d*	0.5
*L*_5_	6.3	*e*	3
*L*_6_	2.72	*f*	2
*L*_7_	1.8	*g*	0.25
*L*_*f*_	14.38	*l*	22
*W*_*m*_	45	*L*_*m*_	65
*W*	45	*m*	1.575
*i*	21.2	*j*	20
*k*	9	*l*	8

**Figure 4:**
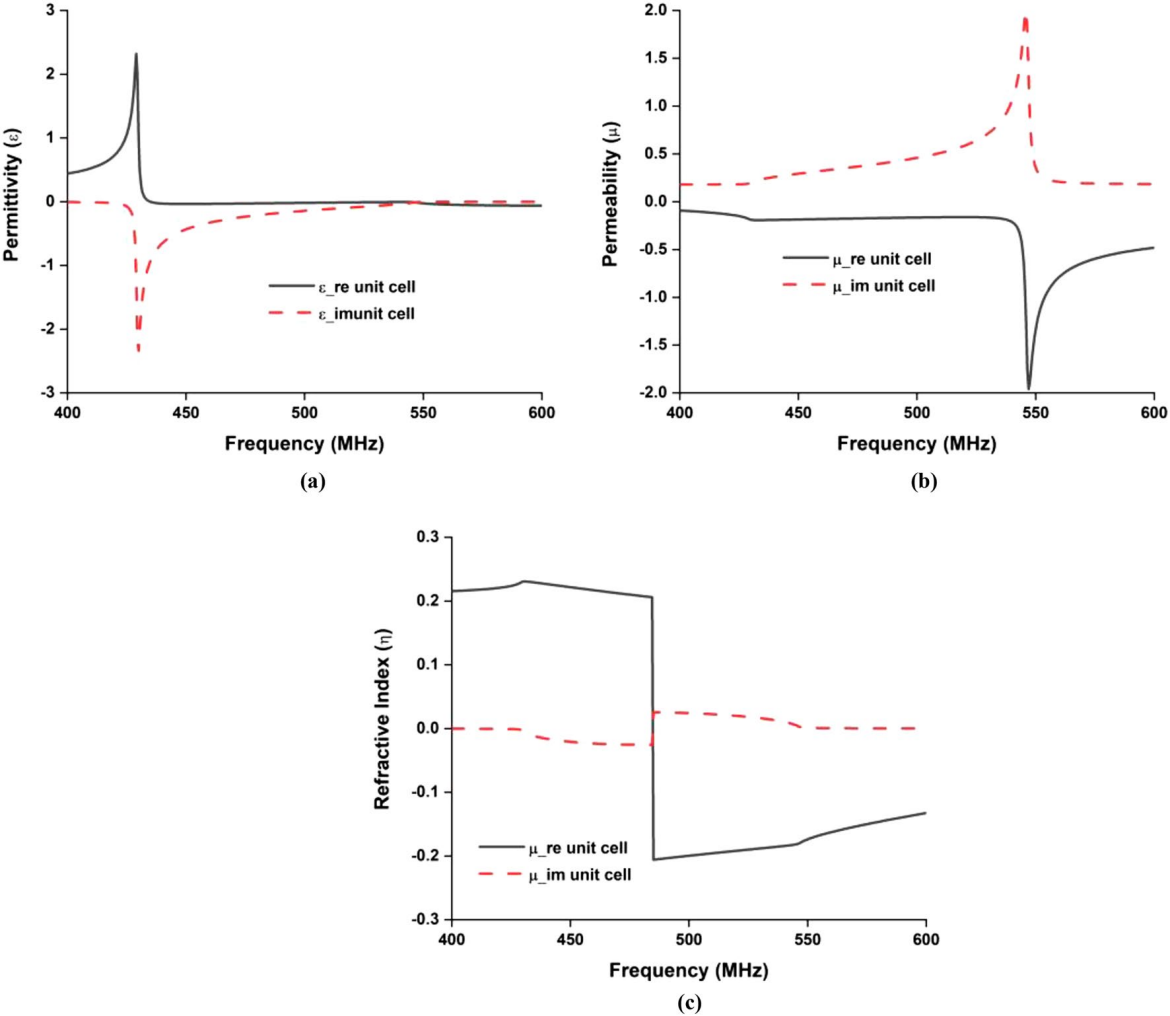
Retrieved metamaterial characteristics for the metamaterial unit cell (**a**) permittivity, (**b**) permeability, and (**c**) refractive index.

**Figure 5:**
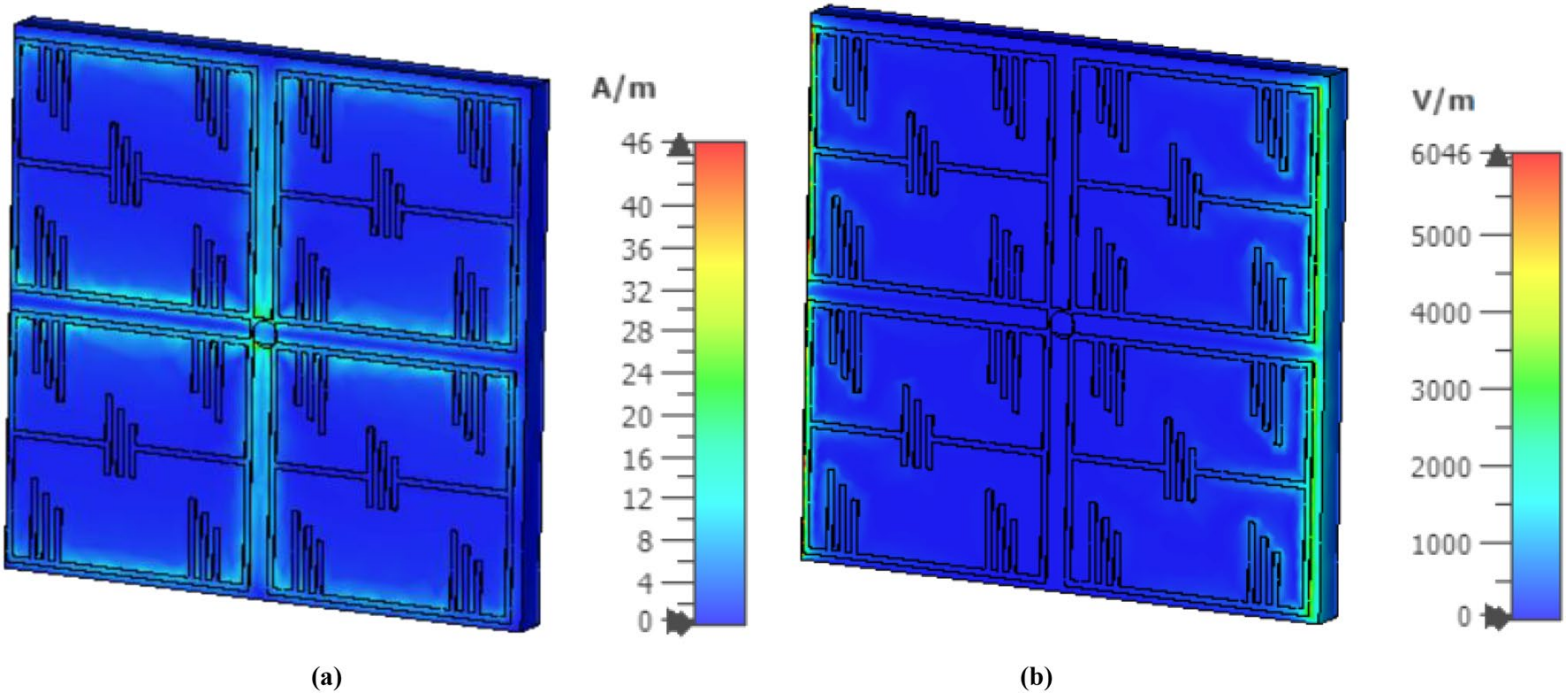
Simulated (**a**) H-field and (**b**) E-field distribution of the designed metamaterial unit cell at 450 MHz (CST STUDIO SUITE 2019, https://www.3ds.com/products-services/simulia/products/cst-studio-suite)^16^.

The metamaterial unit cell layout has been designed, simulated, and investigated using 3D electromagnetic CST microwave studio simulation software. According to^17^, a perfect electric conductor (PEC) in the X-direction and a perfect magnetic conductor (PMC) in the Y-direction has been set as a boundary condition to simulate the metamaterial unit cell structure. Throughout the Z-direction, the electromagnetic wave is propagated. By using the robust methods, the metamaterial unit cell characteristics of permittivity, permeability and refractive index have been investigated after extracting the S parameter from the simulation environment^18, 19^. The unit cell initial dimension has been taken based on the general subwavelength rules as λ/20^20^. Since our target was to design a UHF band 450 MHz metamaterial unit cell, according to the λ/20 relationship, the main dimension of the metamaterial unit cell has taken initially 33 mm. For miniaturization of the unit cell dimension, we have scaled the dimension in CST and introduce some split gap in the unit cell structure, which is described in Fig. 1a. Finally, we have achieved the 22 mm length and width of the split ring resonator of the main unit cell length to achieve. The metamaterial structure depicts near zero permeability (− 0.109), permittivity (0.003) at 450 MHz, shown in Fig. 4. Besides, it is shown from Fig. 4 that the real value of the near-zero (NZ) permeability of the unit cell structure show from 400 to 540 MHz, NZ permittivity shows from 433 to 600 MHz and the near-zero refractive index shows in the entire operating bandwidth. The H-field and E-field distribution of the designed metamaterial has been examined, which is revealed in Fig. 5. It can be observed from Fig. 5 that the effective epsilon (ε) is achieved towards near-zero (ENZ) with the help of outer metallic arms, whereas effective μ near-zero (MNZ) is obtained due to the magnetic resonance experienced by split-ring resonators due to their interconnection. Therefore, the structure can attain impedance-matched near-zero-index metamaterial (NZIM) characteristics with both ENZ and MNZ properties.

**Figure 6:**
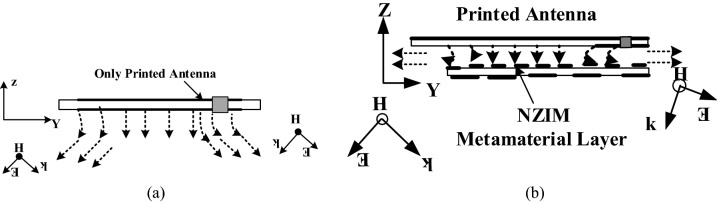
(**a**) Only antenna, (**b**) antenna with EMNZ metamaterial layer.

**Figure 7:**
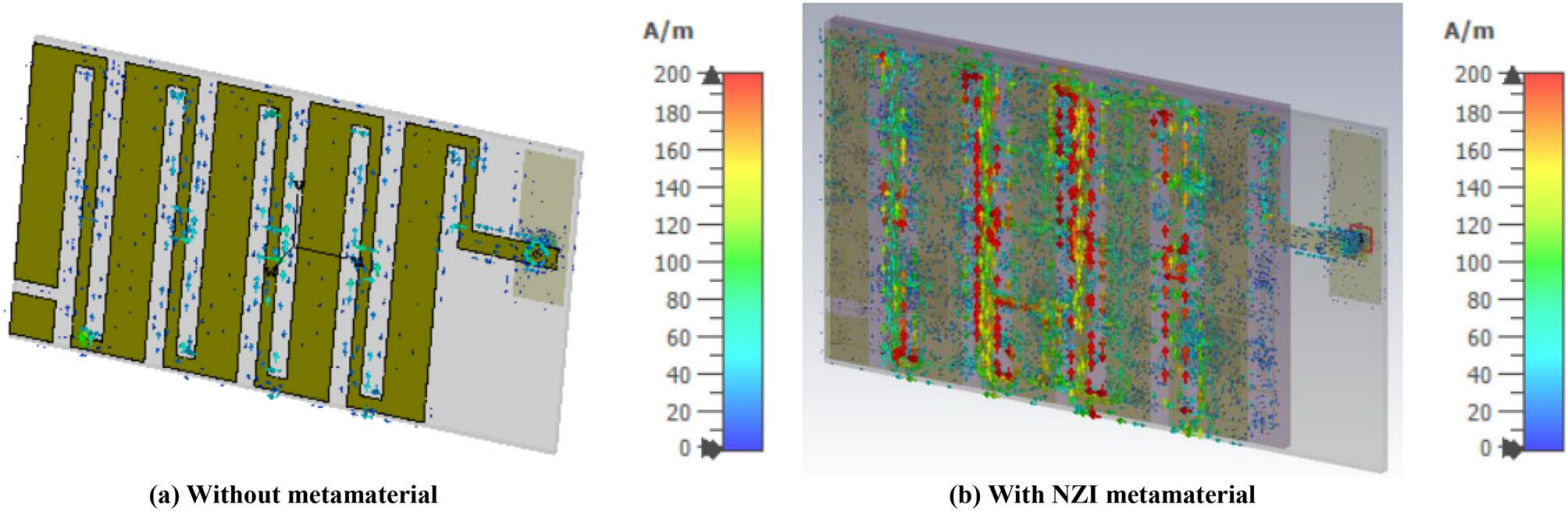
Surface current distribution of the proposed antenna (CST STUDIO SUITE 2019, https://www.3ds.com/products-services/simulia/products/cst-studio-suite)^16^.

The antenna performance enhancement mechanism of the proposed UHF antenna using NZI metamaterial can be explained through Fig. 6a,b. When the sideward electromagnetic wave (EM) wave is incident to the NZRI layer, the sideward radiation power possesses the electromagnetic field E component perpendicular to the cover (along the Z-axis). Due to the mechanism of NZI metamaterial that EM waves are emitted from the NZI layer along the regular direction of the interfaces, some sideward EM waves are parallel to the XY plane after passing through the metamaterial cover. For that reason, the increased EM waves are parallel to the UHF planar antennas horizontal plane, changes the direction of the main beam of the antenna so that the antenna performance like gain and efficiency are increased. Besides, the antenna performance mechanism with and without NZI metamaterial is also investigated by using the surface current analysis which is shown in Fig. 7. It can be observed that after NZI metamaterial incorporation, a strong current is developed near meander line. It can be predicted that the NZI structure has driven the surface current and act as vital radiation elements. Hence, the meander line with metamaterial radiated stronger radiation fields than without a metamaterial antenna and contribute to improving the radiation efficiency and gain of the antenna.

## Experimental results and discussion

**Figure 8:**
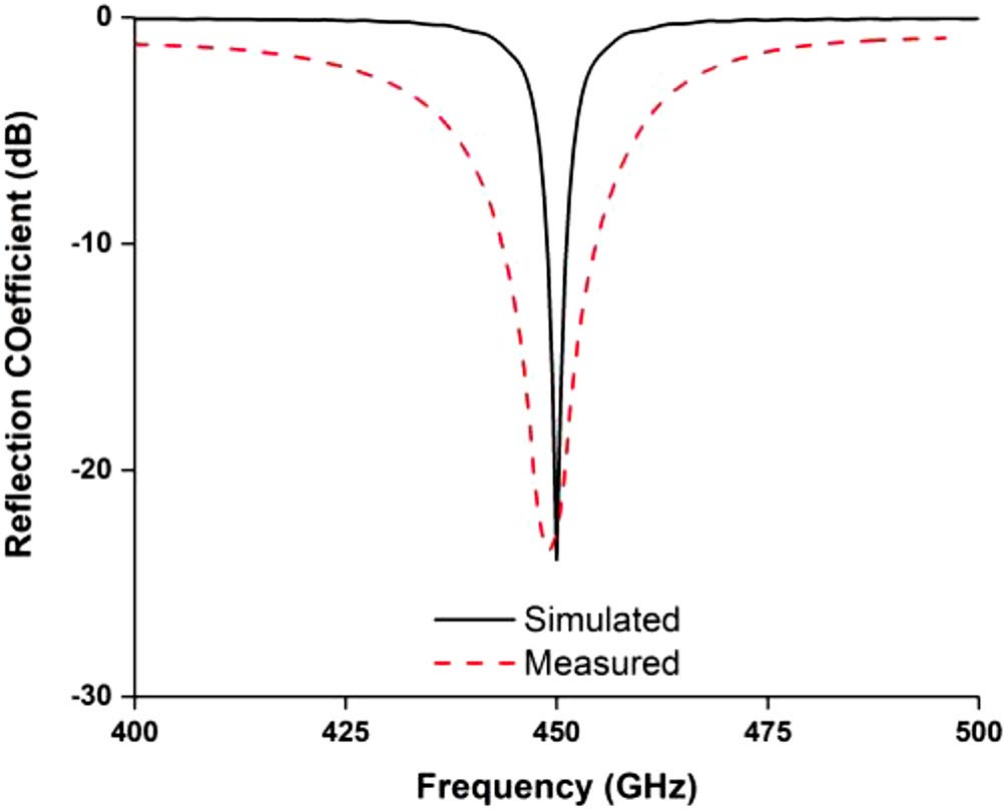
The numerical and experimental reflection coefficient of the optimized antenna.

**Figure 9:**
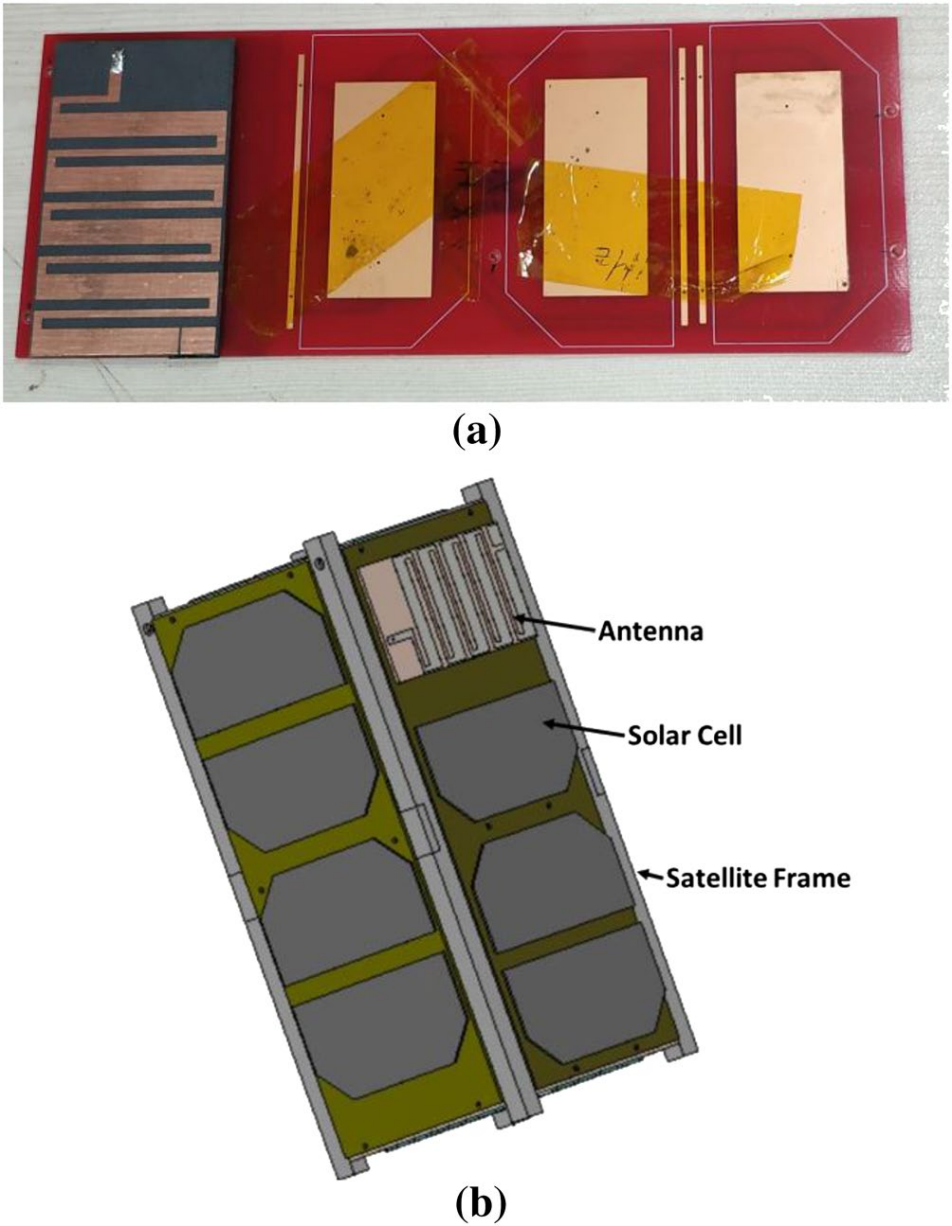
Optimized antenna mounted on 2U Cube satellite body in (**a**) fabricated structure and (**b**) simulation environment.

**Figure 10:**
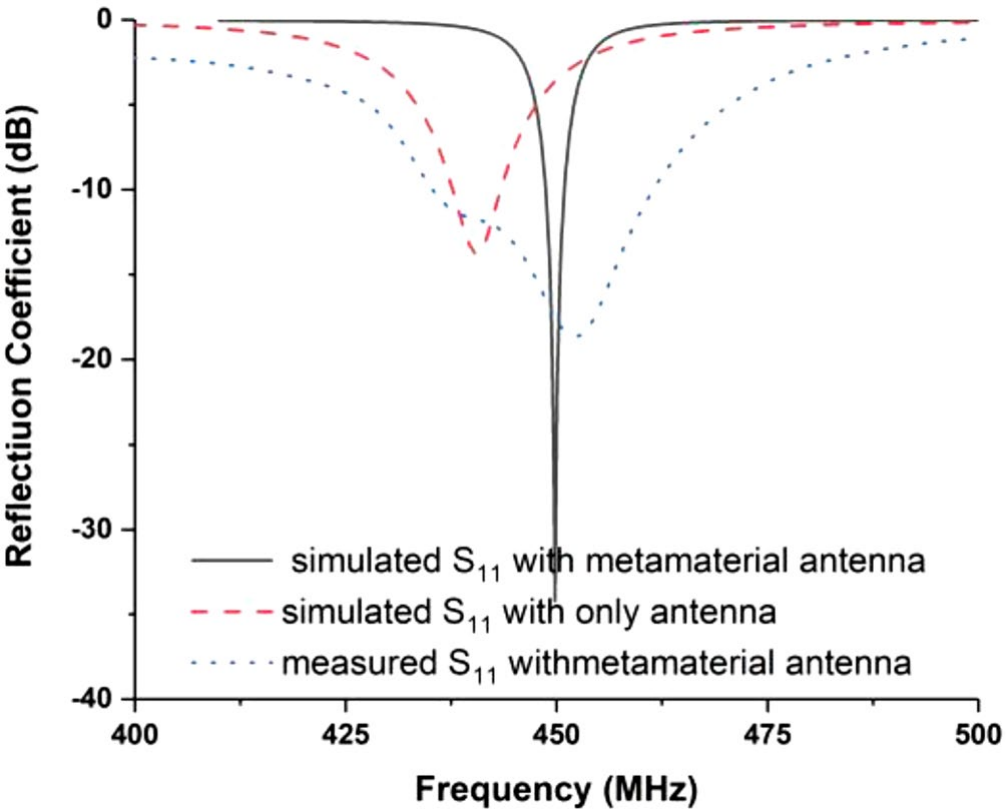
Reflection coefficient (S_11_) of the optimized antenna with 2U Cube Satellite.

**Figure 11:**
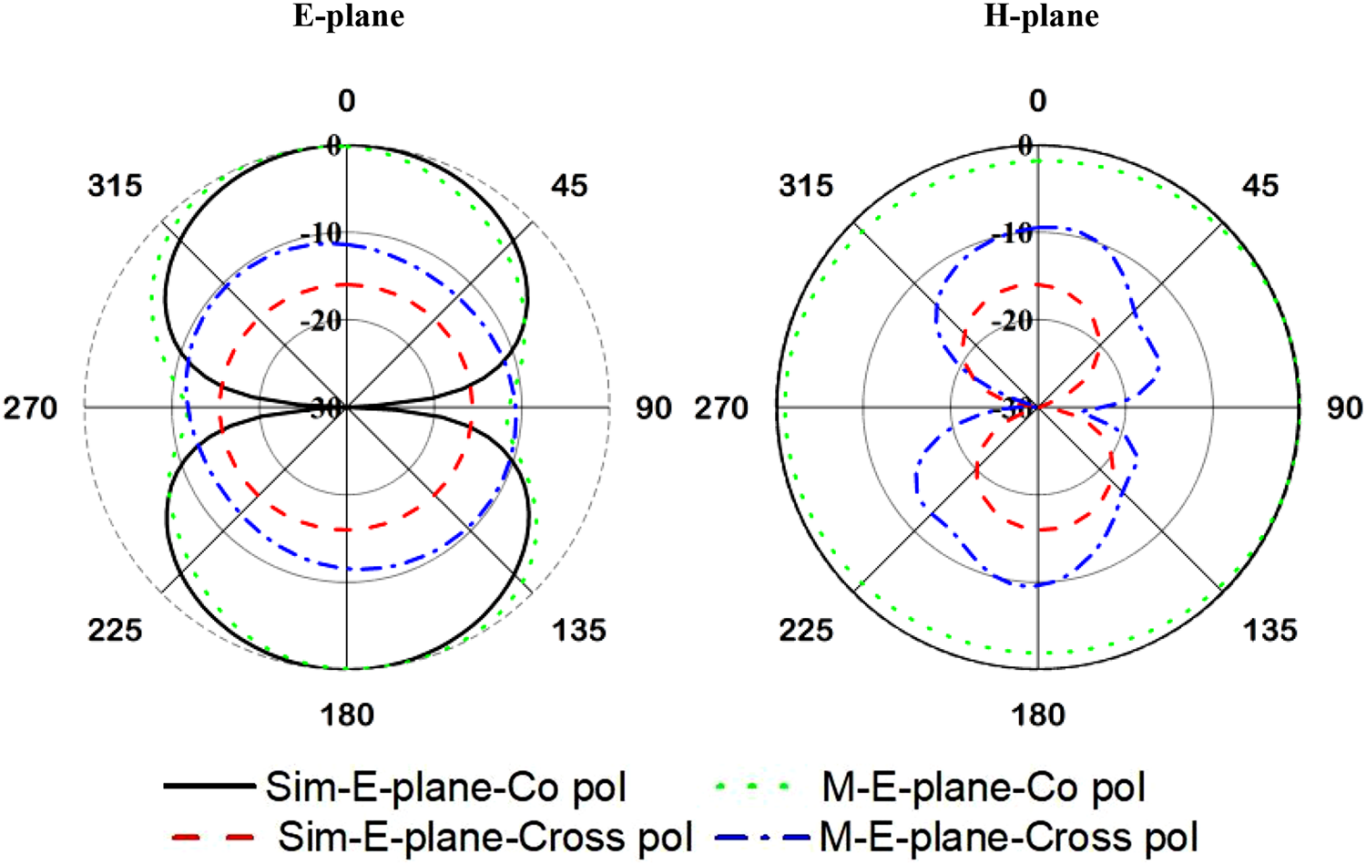
Simulated and measured 2D radiation patterns of the proposed antenna mounted on Cube satellite backplane at 450 MHz.

The final design parameters enlisted in Table 1 have been used for the fabrication of the antenna prototype in a lab environment. The numerical and measured reflection coefficient (S_11_) has been measured in free space, which is shown in Fig. 8. The antenna achieved resonance at 450 MHz in simulation. In measurement, the resonance remains the same position, but the impedance bandwidth increased, which is 443.5–455 MHz. The possible reasons for discrepancy between simulated and measured results is fabrication tolerance and feeding tolerance. Moreover, copper etching, high temperature soldering may have effect on substrate material properties. Besides, as the antenna is fed by a 50 Ω micro-miniature coaxial (MMCX) connector. Therefore, another extension cable and converter are required to connect the antenna with VNA. This extension cable losses during measurement might be another possible reason. These issues were not considered in simulations environment. The antenna performance has also been investigated with a 2U cube satellite body structure, which is shown in Fig. 9. The measured reflection coefficient has been examined while the antenna is mounted on the 2U Cube satellite structure, depicted in Fig. 10. In measurement, the antenna resonance slightly shifted downwards and tuned at 450 MHz, and the antenna shows − 10 dB impedance bandwidth shows from 435.5 to 461 MHz. The peak resonance in simulated and measured results are identical, though a mismatch is observed. In measurement the antenna performance has been investigated using live 2U CubeSat structure. The structure was considered as 227 mm tall in Z axis, 100 mm length in X and Y axes with maximum weight was 2.6 kg. Aluminium 7075 material was considered for the nanosatellite frame structure. The proposed antenna and solar panels have been mounted on the FR-4 backplane oard using RTV glue (Room-Temperature-Vulcanizing glue). The structure has a single motherboard, which is placed inside the structure. Moreover, several subsystem and parts like battery, font panel, transmitter and communication board, payload circuitry etc. have been considered as in its real properties. A dummy mass made of Aluminium 7075 substrate was placed at the middle of the structure to balance the structure. However, in simulation only the outer structure and backplane with solar panel were considered to examine the antenna performances. These factors may be the possible reasons for the discrepancy in simulation and measurement results. The 2D radiation characteristics of the fabricated antenna with the 2U Cube satellite structure have also been examined using the Satimo Starlab Nearfield Measurement System^21^. The StarLab is capable of measuring frequency from 350 MHz to 6 GHz. The baseline configuration is obtained by connecting StarLab to a Vector Network Analyzer (VNA) for passive antenna measurements. The simulated and measured 2D radiation patterns of the antenna with Cube satellite structure are found to be in good agreement in H-plane and maintained approximate omnidirectional radiation pattern, presented in Fig. 11. However, the cross-polarization value has been increased in measurement, and that might occur due to the presence of the metallic structure. Similarly, the E-plane radiation pattern shows little disagreement in measurement. The radiation efficiency and gain of the antenna while integrated with Cube satellite body structure have also been analyzed. The antenna obtained 69% radiation efficiency and gain of 2.5 dB with the Cube satellite structure.

**Figure 12:**
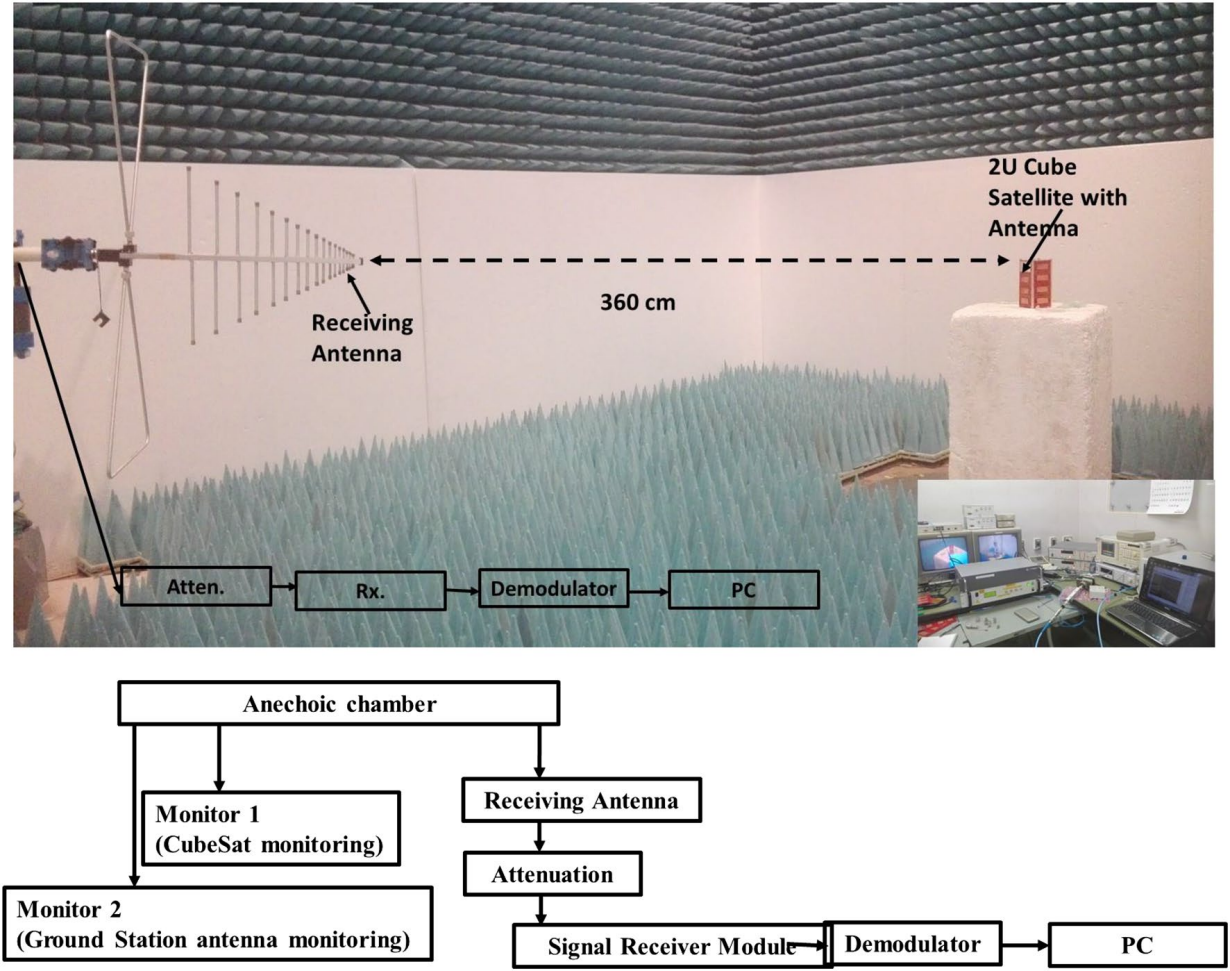
FSPL estimation in the anechoic chamber and maximum attenuation measurement until signal demodulation.

The assessment of signal propagation with maximum value for the proposed antenna is accomplished by conducting free space path loss (FSPL) calculation using variable attenuation. The measurement was performed in the Laboratory of Spacecraft Environment Interaction Engineering (LaSEINE), Kyushu Institute of Technology, Japan. To perform this estimation antenna is incorporated with an active Cube Satellite. LEO orbit at a distance of 400 km from the ground is considered for the calculation purpose of FSPL. Friis transmission equation has been employed for this calculation^22^. The antenna has been tuned at 467 MHz and mounted on a 2U Cube Satellite structure due to measurement limitations that can only measure FSPL at 467 MHz. Active communication board has been integrated into the satellite and connected with the proposed antenna. The antenna is functioned as the transmitting antenna (Tx). The measurement setup is illustrated in Fig. 12. The receiving antenna (Rx) is placed in the horizontal orientation and connected to the receiver module through a variable attenuator. The transmitted signal from the satellite through proposed antenna has been demodulated and investigated the maximum level of attenuation until the smooth demodulation is possible. The maximum level of attenuation represents the signal strength of the transmitter antenna, which can address the maximum path loss.

The free space path loss in an anechoic chamber (FSPL) = 36.95 dB.

The FSPL at an orbital altitude of 400 m = 137.9 dB.

The log periodic antenna gain (G_Rx_) = 6.9 dBi.

The ground station antenna gain (G′_Rx_) = 18 dBi.

The extra attenuation is required to achieve signal level = Orbital FSPL – FSPL − G′_Rx_ + G_Rx_ = 89.85 dB.

It is seen from Table 2 that the proposed antenna facilities 9 dB extra attenuation for signal demodulation.

**Table 2:** Achieved maximum attenuation to address the maximum FSPL.

Cube satellite rotation (angle, degree)	Max. attenuation value (dB)
0	98
20	104.5
80	98
100	98
180	99
200	98
280	98
300	98

A comparison between the proposed UHF cube satellite antenna, commercial antennas and some other existing small satellite antenna papers are given in Table 3. This comparison's main parameters are antenna dimension (mm), antenna type, operating frequency (MHz), realized gain (dBi), cube satellite compatibility and remarkable comments where the proposed antenna performances are better than compared research^6, 23–33^ based on various parameters. From Table 3, it is seen that the proposed antenna provides a significant trade-off between the antenna size and performance in terms of operating frequency, gain, and efficiency for successful CubeSat payload mission. Moreover, the antenna gain integrated with Cube satellite structure is satisfactory for smooth uplink and downlink communication, which is verified in FSFL measurement in Fig. 12. Based on the comparison and CubeSat antenna design constraints, the proposed antenna has achieved potentiality for smooth CubeSat payload operation.

**Table 3:** A comparison of the proposed MTM loaded Patch antenna with existing UHF cube satellite antennas.

Existing research	Antenna dimension (mm)	Antenna type	Operating frequency (MHz)	Realized gain (dBi)	Cube satellite compatibility	Comments
^6^	80 × 45 × 1.575	Planar Patch Antenna Printed EMNZ metamaterial	391–405	1.77	Compatible	Low gain and low efficiency
^23^	320 × 80 × 3.17	Printed Antenna	427–437	2.12	Incompatible	Larger dimension
^24, 25^	72 × 32 × 1.575	Planar Patch Antenna	418–448	0.55	Compatible	Low gain and operating frequency shifted after mount in body
^26^	170 × 120 × 6.4	Microstrip Patch	435–437	0.7	Incompatible	Larger dimension and low gain
^27^	150 × 150 × 37	Microstrip Patch	384–410	0.4	Incompatible	Larger dimension and low gain
^28^	Height :500	Deployable helix	365	8	Incompatible	Antenna performance high but incompatible with 1U CubeSat
^29^	175	Deployable monopole	435–440	0.08	Compatible	Low efficiency
^30^	circumference of a 1.5U CubeSat 100 × 100 × 150	cavity backed slot antenna	485 MHz	2.73 dB	Compatible	–
^31^	92 × 40	Planar patch	435 MHz	-1 dB	Compatible	55%
^32^	85 × 85 × 31	Inverted-F antenna	400 MHz	5.37	Compatible	–
^33^	100 × 100 × 9.4	Stack antenna	2025–2290 MHz	7.4	Compatible	70–96%
^34, 35^	98 × 98 × 7 mm^3^ Envelope stowed 170 mm length	Deployable monopole	435–438 MHz	0	Compatible	–
Proposed design	80 × 40 × 3.35	Planar patch antenna with embedded NZIM layer	443.5–455	2.5	Compatible	69% with realized gain of 2.5 dB at operating frequency

## Conclusion

This research article described the design, simulation, and measurement of a UHF NZI metamaterial antenna for the CubeSatellite communication system. The numerical results are in good agreement with the measurement results. The advantages of the optimized metamaterial loaded antenna are wide impedance bandwidth with stable gain, compact dimension, easy fabrication, and flexibility in designing antenna at desired frequency with optimized the geometrical parameters. The assessment of signal propagation with maximum value for the proposed antenna is accomplished by conducting FSPL calculation using variable attenuation, successfully. In this regard, the proposed NZIM metamaterial embedded compact UHF printed antenna have the potential to be used in LEO orbit Cube Satellite for command function, tracking and data downlink applications.
